# Altered neutrophil glycolysis contributes to microvascular inflammation in an AD mouse model

**DOI:** 10.1002/alz.087124

**Published:** 2025-01-03

**Authors:** Oliver Bracko

**Affiliations:** ^1^ University of Miami, Coral Gables, FL USA

## Abstract

**Background:**

Cerebral blood flow is decreased in mouse models and patients of Alzheimer’s disease (AD). We identified that about 2% of cortical capillaries in the APP/PS1 mouse model of AD had stalled blood flow due to neutrophils obstructing capillaries and contributing to vascular inflammation. Neutrophils are more reactive in AD. Here we are investigating if changes in metabolism contribute to reactive neutrophils driving low‐level chronic vascular inflammation and cerebral blood flow (CBF) reductions in mouse models of AD.

**Methods:**

AD and control neutrophils were isolated and transferred into donor mice. The number of stalled capillaries and blood flow speeds were measured using high‐resolution from *in vivo* two‐photon imaging. Mitochondrial stress and glycolysis were determined using the Seahorse platform.

**Results:**

We found that neutrophils from AD mice caused capillary stalling and contributed to CBF reductions in control mice. Furthermore, we identified that glycolysis is reduced, and mitochondrial stress is increased in AD neutrophils compared to controls. Lastly, the maximal oxygen consumption rate is reduced in AD neutrophils indicating mitochondrial dysfunction.

**Conclusion:**

This data demonstrates that reactive neutrophils in AD mice contribute to capillary stalling and CBF reductions. Glucose metabolism is reduced in neutrophils, indicating a metabolic shift from aerobic to anaerobic glycolysis. Overall, the data shows that neutrophils are more reactive at baseline in AD mice and likely contribute directly to chronic inflammation seen in mouse models and patients with AD.

